# Carryover Effects of Thermal Conditions on Tick Survival, Behavior, and Simulated Detectability

**DOI:** 10.1002/ece3.72252

**Published:** 2025-10-02

**Authors:** Daniel S. Marshall, Karen C. Poh, Jeb P. Owen

**Affiliations:** ^1^ Department of Entomology Washington State University Pullman Washington USA; ^2^ Animal Disease Research Unit, USDA ARS Pullman Washington USA

**Keywords:** abiotic effects, *Amblyomma americanum*, behavior, hazard, risk, vector‐borne disease

## Abstract

Carryover effects occur when environmental history of an organism influences its behavior, fitness, and population dynamics. Carryover effects have received some attention in the field of vector‐borne disease ecology but are understudied in the context of ticks and tick‐borne pathogen transmission where they may influence tick–host contact, pathogen transmission, and tick surveillance. Using controlled lab studies, we investigated how recent thermal history affects mortality and activity of adult *Amblyomma americanum* , an emerging vector of human and animal pathogens. To characterize thermal carryover effects on tick detection, we used our laboratory data to parameterize a simulation of tick trapping in the field. Ticks exposed to warm conditions for 4 weeks subsequently exhibited an increased mortality rate and heightened activity levels (as measured by time spent moving and distance moved in 24 h) that declined over time compared to ticks with cool thermal histories that had a lower mortality rate and maintained steady activity levels over time. Past thermal conditions had carryover effects on tick detection with simulated trapping. Early in the simulation (Days 0–8) ticks with a warm history were detected at higher rates due to carryover effects on tick movement. Later in the simulation (Days 10–20) ticks with a cool history were detected at higher rates due to a combination of carryover effects on movement and mortality. These findings demonstrate short‐term thermal carryover effects on adult *A. americanum* that have implications for tick surveillance and tick‐borne pathogen risk.

## Graphical abstract

Recent thermal history affects the abundance and behavior of adult *Amblyomma americanum* . Thermal responses impacted simulated tick detection through combined effects on movement and mortality.

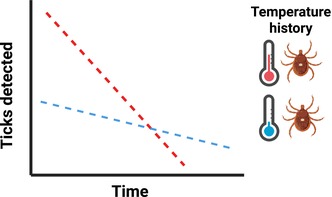

## 1 Introduction

Ticks are obligate, blood‐feeding ectoparasites that are of broad concern to human and animal health worldwide (Jongejan and Uilenberg 2004). Understanding the environmental factors that affect tick abundance and behavior remains an important research objective because tick‐borne pathogen transmission is density‐dependent (Porter et al. 1988; Walk et al. 2009; Van Gestel et al. 2024) and influenced by host‐seeking behavior (Falco et al. 1999; Cat et al. 2017; Van Gestel et al. 2024). Many tick species spend long periods of time off‐host in the open environment (Oliver 1989). During these off‐host periods, a tick must move to avoid unfavorable environmental conditions (Bertrand and Wilson 1996; Crooks and Randolph 2006), maintain water balance (Rudolph and Knülle 1974; Crooks and Randolph 2006), and position itself to come in contact with hosts (Randolph and Storey 1999; Burton et al. 2024) while conserving energy between blood meals (Oliver 1989). Considerable work has focused on the interaction between weather conditions, tick survival, and behavior. Tick movement and time spent foraging change with ambient temperature (Vail and Smith 2002; Benoit et al. 2021; Fieler et al. 2021), which alters the probability of contact with hosts (Duffy and Campbell 1994; Schulze et al. 2001; Burtis et al. 2016). Ambient temperatures also strongly affect tick longevity (Koch and Tuck 1986; Wilson et al. 1993; Zahler and Gothe 1995; Troughton and Levin 2007; Owen et al. 2014; Nielebeck et al. 2023), which alters the abundance of host‐seeking ticks in the environment. Importantly, across these studies, tick behavior and survival have been assessed under concurrent abiotic conditions. With few exceptions, the impact of past conditions on tick traits (i.e., carryover effects) has not been studied (Ogden et al. 2004; Sutherst and Bourne 2006).

Carryover effects have a long history of study in clinical medicine (O'Connor et al. 2014) and, more recently, in the field of ecology (Harrison et al. 2011; O'Connor et al. 2014). A carryover effect occurs when prior conditions affect an individual's current performance (O'Connor et al. 2014). Individuals in a population may have experienced different environmental conditions or nutritional inputs (Harrison et al. 2011). These past conditions can produce varied carryover effects on fitness (Festa‐Bianchet 1998; Gunnarsson et al. 2005), population dynamics (Norris 2005; Norris and Taylor 2006), and community structure (Gratton and Denno 2003; Van Allen and Rudolf 2016). Among the few studies of carryover effects on ticks, there is evidence that past ambient temperature influences behavior, development, survival, and reproduction. Past exposure to cold temperatures slows subsequent development and oviposition (Ogden et al. 2004) and influences mortality rates (Sutherst and Bourne 2006). Thermal history can also change foraging behavior. *Ixodes ricinus* from cooler climates initiate questing at lower temperatures than those from warmer ones (Gilbert et al. 2014). Although robust data illustrate effects of concurrent temperature on tick ecology, it remains unclear how the abundance or behavior of ticks may be shaped by past weather or how current conditions may influence ticks in the future.

Knowledge of environmental effects on ticks is relevant to tick surveillance and forecasts of tick‐borne pathogen transmission (Falco et al. 1999; Cat et al. 2017; Van Gestel et al. 2024). Tick surveillance is conducted using methods that actively collect ticks from vegetation (i.e., drags) or attract ticks to traps with carbon dioxide (Schulze et al. 1997; Eisen and Paddock 2021). These methods provide counts of ticks, which are used to estimate tick abundance or host‐seeking activity in areas of habitat. Only ticks actively questing on vegetation will be detected by drags, and only ticks responsive to baits (e.g., CO_2_) will move to a trap (Falco and Fish 1991; Schulze et al. 1997; Kensinger and Allan 2011). The number of ticks detected is positively associated with the abundance of ticks in the environment, but traps and drags only detect a fraction of the ticks that are present (Dobson 2014; Sirén et al. 2024; Marshall et al. 2025) because only a subset of ticks in an area are questing or responsive to baits at any given time (Lees and Milne 1951; Jensen 2000; Marshall et al. 2025). This creates a fundamental problem with tick surveillance—the detected abundance of ticks may fluctuate as a function of changes in the number of ticks present and as a function of the activity levels among the ticks present. Although abiotic conditions affect tick survival and behavior, it is unclear how these parameters jointly affect the detection of ticks.

For this study, we explored how temperature conditions influence the likelihood of tick detection through the lens of carryover effects. We aimed to characterize how past temperature conditions influence mortality and movement (i.e., activity), followed by modeling the joint effects of mortality and movement on simulated tick detection. We based our experiments on *Amblyomma americanum* —a highly mobile and widespread tick in the eastern United States (Raghavan et al. 2019). Based on published work with hard ticks (van Es et al. 1998; Oyen et al. 2021; Nielebeck et al. 2023), we hypothesized that increased ambient temperatures would result in increased tick activity and decreased survival, which would alter the number of ticks trapped (detected). We discuss our results in the context of the behavioral ecology of *A. americanum* , which is responsible for transmitting numerous pathogens to humans and other animals (Hopla 1960; Barnard 1985; Mixson et al. 2004; Savage et al. 2013, 2017; Levin et al. 2017) and continues to increase its geographic range, potentially exposing naïve populations to pathogens (Springer et al. 2014; Stafford III et al. 2018; Raghavan et al. 2019).

## 2 Materials and Methods

### 2.1 Environmental Conditions

Adult *A. americanum* were acquired from a laboratory colony maintained by Oklahoma State University. Ticks belonged to a single cohort (i.e., blood fed as nymphs and molted to the adult stage at the same time) which molted on 25 September 2023. Upon receipt from Oklahoma State University (approximately 2 months post‐molt), adult ticks were split into two groups maintained in incubators at either high ambient temperature (“warm history,” 35°C; *N* = 96) or low ambient temperature (“cool history,” 10°C; *N* = 96). Both temperature conditions were kept at 90% relative humidity and 14:10 light: dark (diel cycle; 14 h light and 10 h dark). These conditions reflect moderate to high temperatures in Oklahoma between early spring and late fall when *A. americanum* are active (Cobos et al. 2024). Ticks remained in these conditions for 4 weeks after which they were placed into one of two common environmental chambers. For the behavior experiment (see below), we placed ticks (*N* = 46 warm history, *N* = 46 cool history) into an environmental chamber with conditions of 10°C, 90% relative humidity, and 14:10 light: dark. We expected these cooler conditions to reduce mortality and help maintain enough ticks for behavioral observations. To conduct the survivorship experiment (see below), ticks (*N* = 30 warm history, *N* = 30 cool history) were placed into an environmental chamber with conditions of 40°C, 90% relative humidity, and 14:10 light: dark. These conditions fall at the upper end of weather experienced by *A. americanum* in Oklahoma (Cobos et al. 2024) and were expected to cause mortality over the course of a month (Nielebeck et al. 2023).

### 2.2 Behavior Experiment

After 4 weeks in fixed environmental conditions, both warm history (*N* = 46) and cool history (*N* = 46) *A. americanum* were moved into a common environmental chamber (10°C, 90% relative humidity, 14:10 light:dark) for 1–35 days before bioassay of movement behavior. Our bioassay design allowed for four tick observations per 24 h. For bioassays, ticks were removed from the common environment and individually placed under a video recording setup as previously described in Marshall et al. (2025). Briefly, individual ticks were moved into separate glass petri dishes set on a light box to “under light” each tick from below. The light box was constructed from an aquarium turned on its side that was lined with sheets of mylar to diffuse light and was lit from the interior using a LED ring light (Figure S1).

For a bioassay replicate, four ticks were video recorded simultaneously, with one tick per petri dish. Both warm and cold history ticks (*N* = 2 of each treatment) were observed in each recording replicate using a digital camera (Canon EOS Rebel T100, Canon Inc., Ōta, Japan) that was positioned above the Petri dishes and oriented downward (i.e., viewing underlit ticks from above). Equal numbers of ticks from each group were tested simultaneously over the 35‐day period to prevent bias among the two groups in the time elapsed since their respective temperature treatments. We ensured that camera focus and field of view were consistent across recordings. Tick movement was recorded for 24 h in a dark room absent of host stimuli and kept at approximately 24°C and 60% relative humidity. Petri dishes were cleaned prior to each observation using hot water with dish soap, then rinsed with 75% ethyl alcohol. EthoVision software (Noldus Information Technology, Wageningen, Netherlands) was used to quantify distance moved and activity time over each 24‐h period.

### 2.3 Survivorship Experiment

After 4 weeks in fixed environmental conditions, warm history (*N* = 30) and cool history (*N* = 30) *A. americanum* were moved into a common environmental chamber (40°C, 90% relative humidity, 14:10 light:dark) and were monitored daily for survival until all ticks were dead. Individual ticks were held in 5 mL centrifuge tubes with perforated caps to allow gas and moisture exchange with the environmental chamber. Ticks were checked every other day for survival. Ticks that did not respond to investigators' breath and handling were deemed dead. The survivorship experiment continued until all ticks died.

### 2.4 Replication Statement

**Table:** 

Scale of inference	Scale at which the factor of interest is applied	Number of replicates at the appropriate scale
Individual	Chambers	46 of each treatment

### 2.5 Statistical Analyses

To analyze the distance moved relative to environmental conditions (i.e., treatment), tick sex, and time elapsed since treatment (i.e., retention time), we used a Gaussian family generalized linear model (distance moved ~ treatment + sex + sex * treatment + retention time * treatment). For time spent moving, we used a Gaussian family generalized linear model (time moving ~ treatment + sex + sex * treatment + retention time * treatment). We performed Kaplan–Meier survivorship analyses with a Cox proportional hazards model to determine the effects of environmental condition and sex on survivorship. Statistical analyses were performed in R (R Core Team 2023) using the *lme4* (v. 1.1–29) (Bates et al. 2015) and *survival* (v. 3.5–8) (Therneau et al. 2024) packages.

### 2.6 Detection Model

The number of trapped ticks (i.e., ticks detected) is influenced by the number of ticks alive on the day of trapping and tick movement to the trap. To investigate how past temperature might affect tick detection, we simulated the joint effects of mortality and movement on surveillance trapping. We assumed that movement is positively associated with probability of trapping, based on the expectation that the further a tick moves, the more likely it is to reach a CO_2_ baited trap. We converted movement distances to probabilities of trapping and simulated the numbers of ticks trapped using a binomial function based on tick abundance (number of trapping trials) and the probability of successful capture derived from tick movement. Carryover effects of temperature on mortality and movement were included in simulated trapping, which allowed us to compare tick detection among simulated tick populations with different past temperatures. Based on measured carryover effects of temperature on mortality, we first simulated abundances of ticks over time. Warm history tick abundance on day *x* ($N_{\mathrm{day}\;x}$) was modeled as a linear function:

$$N_{\mathrm{day}\;x}=N_{\mathrm{day}\;0}+\beta_{\text{condition}}\times {\mathrm{day}}_{x}$$

Mortality rate ($\beta_{\text{condition}}$), which was estimated from lab experiments, was drawn from a normal distribution for warm conditions (mean = −1.71, SD = 0.2). The survivorship curve for ticks with a cool history was distinctly sigmoidal. Thus, cool history tick abundance was modeled as a sigmoid function:

$$N_{\mathrm{day}\;x}=\frac{N_{\mathrm{day}\;0}}{1+e^{-\frac{{\mathrm{day}}_{x}-{\mathrm{day}}_{i}}{\beta_{\text{condition}}}}}$$

Mortality rate $\left(\beta_{\text{condition}}\right)$ and the day on which mortality was steepest $\left({\mathrm{day}}_{i}\right)$ followed normal distributions for cold conditions ($\beta_{\text{condition}}$ mean = −3.0, SD = 0.3; ${\mathrm{day}}_{i}$ mean = 24, SD = 1). Plots of simulated abundance values aligned with observed values (Figures S2 and S3). Next, we simulated tick movement distances over time. Tick movement distance on day *x* ($M_{\mathrm{day}\;x}$) was modeled as:

$$M_{\mathrm{day}\;x}=M_{\mathrm{day}\;0}+\delta_{\text{condition}}\times {\mathrm{day}}_{x}$$

Day zero distance values ($M_{\mathrm{day}\;0}$), which differed for ticks from warm versus cool conditions, were drawn from normal distributions for warm conditions (mean = 150, SD = 10) and cool conditions (mean = 100, SD = 10). Slopes for the change in distance moved ($\delta_{\text{condition}}$) among ticks from warm conditions were drawn from a normal distribution (mean = −2.857, SD = 0.4). For ticks from cool conditions, slopes were set to zero because we did not observe any change in distances moved over time among ticks from cool conditions. Plots of simulated distance values aligned with observed values (Figure S3). Next, we converted movement distances into estimates of trapping probability ($P_{\text{trapped}}$), based on the expectation that the farther a tick moves the more likely it will reach the trap. We treated the relationship between distance moved ($M_{\mathrm{day}\;x}$) and trapping probability ($P_{\text{trapped}}$) as a saturating function (Figure S4):

$$P_{\text{trapped}}=M_{\mathrm{day}\;x}/\left(350+M_{\mathrm{day}\;x}\right)$$

Based on previous mark–recapture field studies and laboratory measures of *A. americanum* movement (Marshall et al. 2025), we adjusted the function to provide a mean probability of trapping equal to 0.3. Finally, to simulate tick detection relative to past temperature conditions, we modeled tick trapping using a binomial distribution with the number of trials drawn from simulated abundances of ticks ($N_{\mathrm{day}\;x}$) and the probability of success on each trial drawn from the simulated probabilities of trapping ($P_{\text{trapped}}$). Detection modeling was performed in R (see File S1 for an R Markdown file).

## 3 Results

### 3.1 Behavior Experiment

**FIGURE 1:**
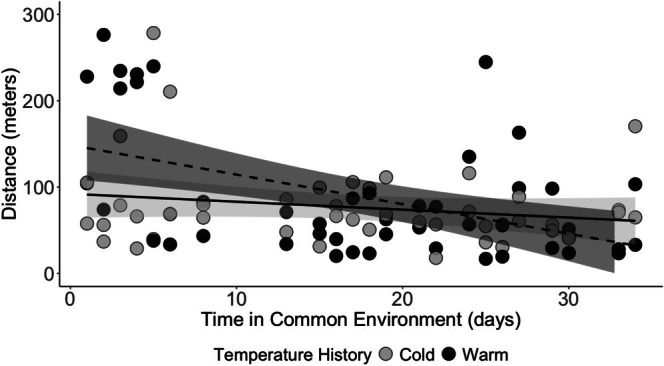
Total distance traveled in 24 h by adult *Amblyomma americanum* ticks with warm (black circle) or cool (gray circle) thermal histories relative to the days elapsed since transfer to a common environment (time in common environment). Trends in movement distances are illustrated by linear fits for warm (dashed) and cool (solid) thermal histories, with 95% confidence intervals indicated by shading.

**FIGURE 2:**
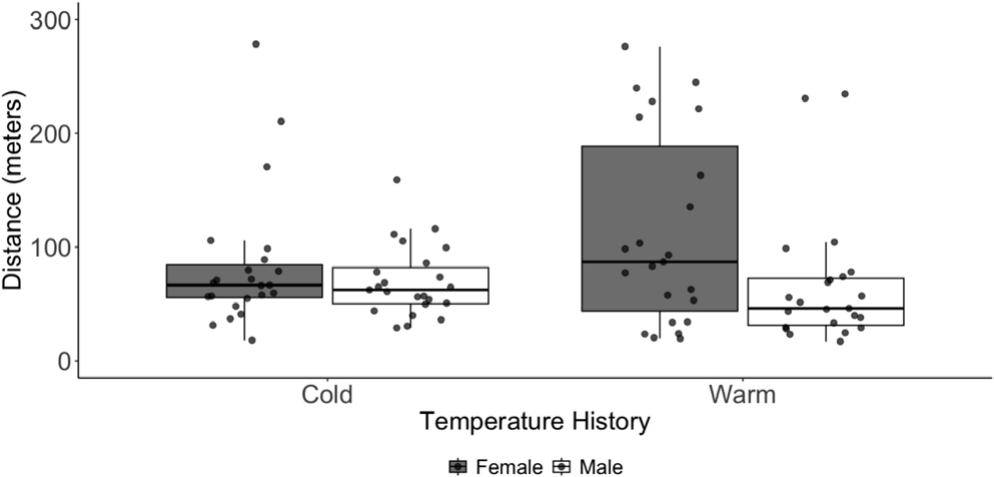
Boxplot of distances moved by male (open box) and female (gray box) laboratory‐reared *Amblyomma americanum* with warm and cool temperature histories. Movement of each tick was measured over a single 24‐h period within 35 days after transfer to a common environment.

We observed a significant interaction between prior ambient temperature and tick movement over time (*t* = −2.193, *p* = 0.031). Ticks from a previously warm environment moved greater distances early after transfer to the common environmental conditions, but movement distances declined at a rate of 3.4 m per day (Figure 1). In contrast, the distances moved by ticks from a previously cool environment were stable over time. There was no significant difference in distances traveled between sexes (*t* = −0.839, *p* = 0.404). Likewise, the interaction of treatment and sex did not affect the distance traveled (*t* = −1.400, *p* = 0.165). The mean (range) distance traveled was 112.7 (19.5–276.3) m (warm females), 66.1 (17.0–234.6) m (warm males), 83.3 (18.0–278.4) m (cold females), and 69.4 (29.0–159.1) m (cold males; Figure 2).

**FIGURE 3:**
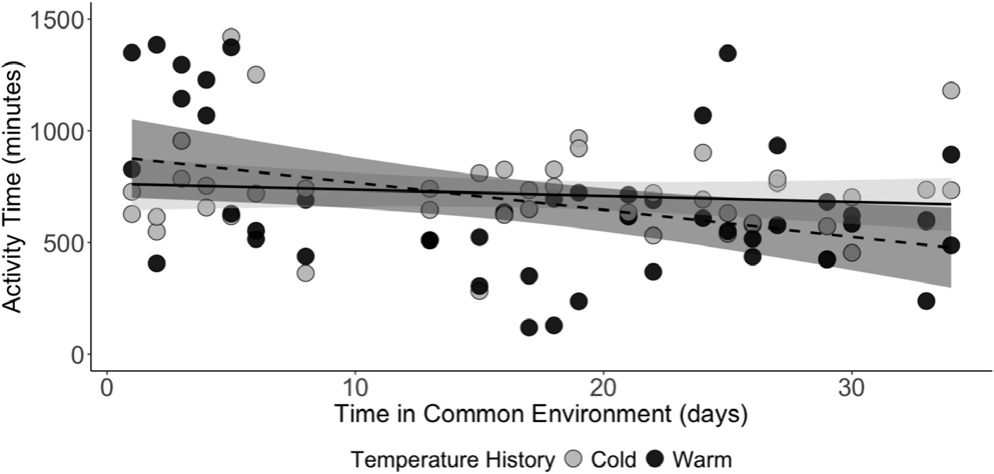
Time spent moving in 24 h by adult *Amblyomma americanum* ticks with warm (black circle) or cool (gray circle) thermal histories relative to the days elapsed since transfer to a common environment (time in common environment). Trends in movement time are illustrated by linear fits for warm (dashed) and cool (solid) thermal histories, with 95% confidence intervals indicated by shading.

**FIGURE 4:**
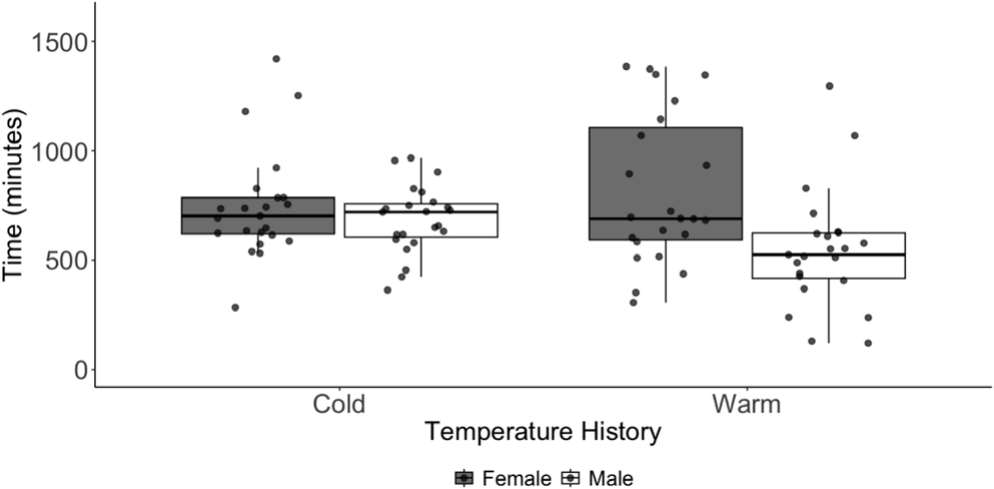
Boxplot of time spent moving by male (open box) and female (gray box) laboratory‐reared *Amblyomma americanum* with warm and cool temperature histories. Movement of each tick was measured over a single 24‐h period within 35 days after transfer to a common environment.

We observed a trending interaction between prior ambient conditions and the duration of movement over time (*t* = −1.842, *p* = 0.069; Figure 3). Ticks from a previously warm environment spent more time moving early after transfer to the common environmental conditions, but the duration of movement declined at a rate of 12 min per day (Figure 3). In contrast, the duration of movement by ticks from a previously cool environment declined at a rate of 2.7 min per day. We observed a significant interaction of treatment and sex on the duration of movement (*t* = −2.021, *p* = 0.046). The mean (range) duration of movement was 815.9 (305.5–1385.3) minutes (warm females), 542.7 (120.0–1295.8) minutes (warm males), 747.8 (283.1–1420.1) minutes (cold females), and 685.2 (363.1–967.0) minutes (cold males; Figure 4).

### 3.2 Survivorship Experiment

**FIGURE 5:**
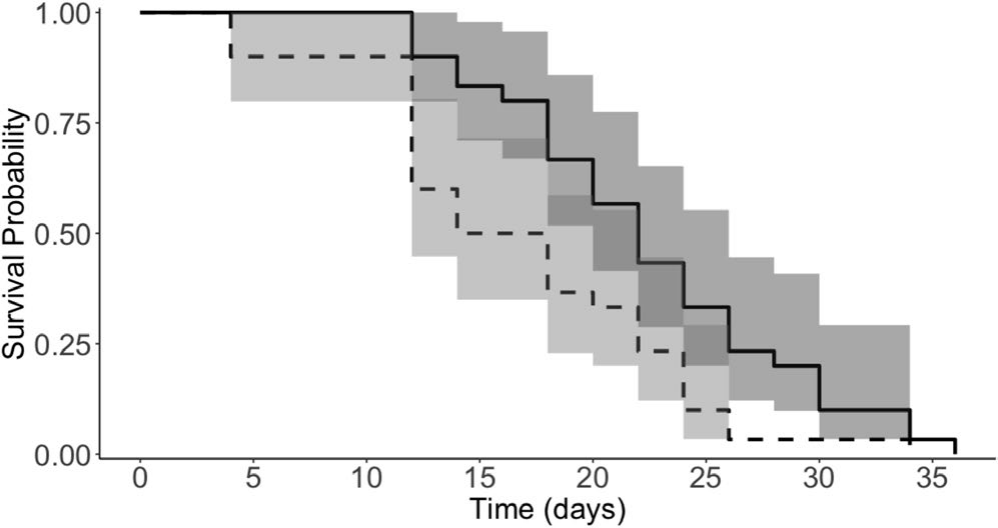
Kaplan–Meier plots of adult *Amblyomma americanum* survival for ticks with warm (dashed line) and cool (solid line) temperature histories. Tick survival was monitored daily after ticks were transferred to a common environment. Shaded areas represent 95% confidence intervals for the estimated survival probability at a given time point.

**FIGURE 6:**
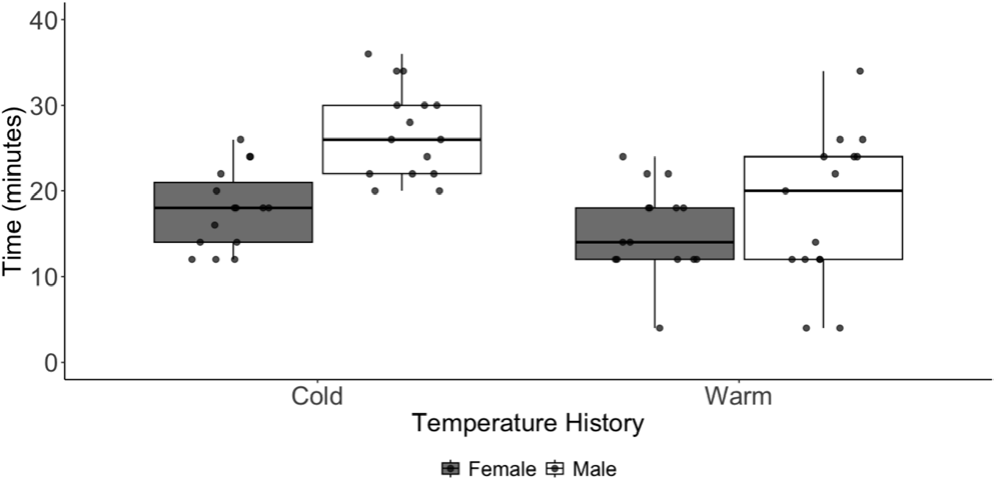
Boxplot of survival times of male and female *Amblyomma americanum* with warm and cool temperature histories. Ticks were moved from warm or cold conditions to common hot environmental conditions (40°C, 90% relative humidity, 14:10 light:dark), and mortality was recorded daily until all ticks had died.

We observed a significant effect of prior ambient temperature on survivorship under hot environmental conditions (*z* = 2.579, *p* = 0.00991) with ticks from previously cool conditions living on average 5.7 days longer than ticks from previously warm conditions. The mean (range) survival time was 16.7 (4–34) days for ticks from the warm history and 22.4 (12–36) days for ticks from the cool history (Figure 5). Tick sex had a statistically significant effect on survivorship, with males surviving longer than females (*z* = −3.895, *p* = 0.0001). The mean (range) survival time was 15.5 (4–24) days for warm females, 18.0 (4–34) days for warm males, 17.9 (12–26) days for cold females, and 26.9 (20–36) days for cold males (Figure 6).

### 3.3 Detection Model

**FIGURE 7:**
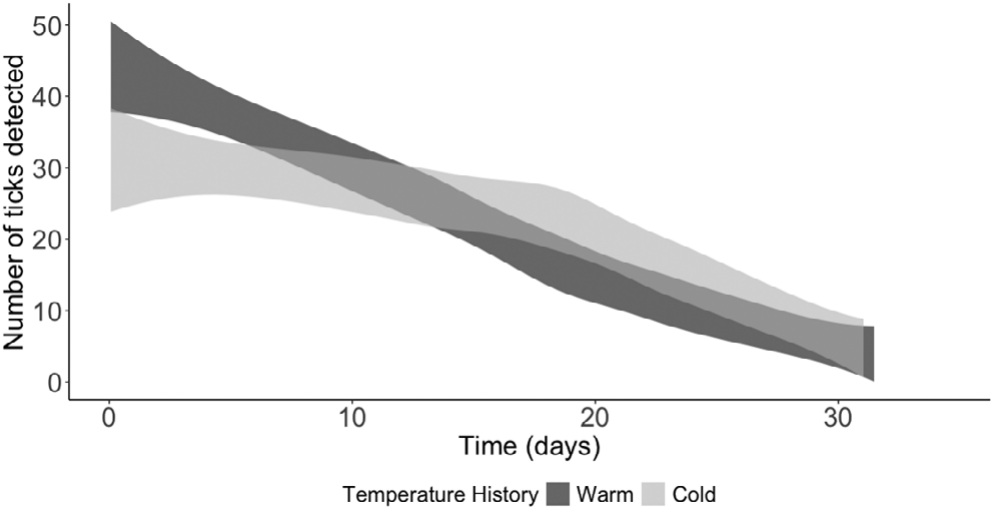
Numbers of detected ticks from simulated trapping events are shown for adult *Amblyomma americanum* with warm (dark gray) and cool (light gray) temperature histories. Trapping events were simulated using random numbers drawn from a binomial distribution, with the number of trapping events reflecting the number of ticks alive at a given timepoint and the probability of trapping based on tick movement distance. Shaded envelopes depict 99% confidence intervals derived from regression fits to simulated data.

Simulated numbers of trapped (detected) ticks declined over a 35‐day period, but the relative numbers of detected ticks differed between thermal histories (Figure 7). Early in the simulation period (Days 0–8), more ticks were detected from the warm history, though the two groups had similar abundance values during that period (Figure S4). Higher distances traveled by warm history ticks (Figures S5 and S6) translated into higher probabilities of detection (Figures S7 and S8). Later in the simulation period (Days 10–20), more ticks were detected from the cool history. This was due to a combination of higher abundances and higher distances traveled among cool history ticks.

## 4 Discussion

Environmental conditions are known to affect tick survival and behavior (Semtner and Hair 1973; Koch 1984; Schulz et al. 2014; Zając et al. 2023), which influence the hazard for tick contact and tick‐borne pathogen transmission (Barnard 1991; Nuttall and Labuda 1994; Van Gestel et al. 2024). Although climate effects have been characterized for concurrent climate conditions, few studies have investigated carryover effects. We examined the delayed effect of temperature on tick mortality rate and movement using a laboratory colony of *A. americanum* . Following 4‐week exposure to warm versus cool ambient temperatures, we monitored survival of ticks and quantified carryover effects on tick mortality rate. Ticks previously exposed to a higher temperature died at a rate 14% greater than ticks previously exposed to a cool temperature. Movement (time and distance) of ticks previously exposed to higher temperatures was initially greater compared to ticks with a cool temperature history, but their movement declined over time. In contrast, movement of ticks with a cool history remained relatively consistent over time. These results suggest that temperature variation in the wild may have carryover effects on both abundance (number alive) and behavior of adult *A. americanum* .

Previous studies have shown evidence of carryover effects on ticks. Exposure of *I. scapularis* females to cold temperatures increased the subsequent preoviposition period measured under warm conditions (Ogden et al. 2004). Exposure of *Rhipicephalus microplus* eggs to cold temperatures or desiccation stress increased subsequent mortality rates of hatched larvae (Sutherst and Bourne 2006). Our data show that 1 month of exposure to temperatures at the lower and upper ends of the natural range has carryover effects on adult *A. americanum* survival and movement under common environmental conditions over a 35‐day period. We observed increased mortality among warm history ticks relative to those with a cold temperature history. It is unknown how thermal conditions produce carryover effects on mortality rate, but the literature provides evidence of several possible mechanisms. Higher ambient temperature may have increased the tick's metabolic rate and demand on limited energy reserves (Halsey et al. 2015). This could have caused warm history ticks to have lower energy reserves when placed in the common environment, resulting in more rapid mortality due to starvation (Rosendale et al. 2017). Similarly, despite stable relative humidity between treatments, warm history ticks may have become water stressed and used energy reserves to generate metabolic water, expending more energy reserves relative to cold history ticks (Rosendale et al. 2016).

The impact of thermal carryover effects on tick survival is relevant to tick‐borne pathogen transmission because transmission is dependent on tick abundance (Barnard 1991; Pepin et al. 2012; Walter et al. 2016). Put simply, higher numbers of ticks increase the potential for tick–host contact and transmission events. Thus, carryover effects on tick mortality rate could alter the risk of pathogen transmission by affecting the abundance of host‐seeking ticks over time. However, the probability of tick–host contact is also strongly affected by tick behavior (Duffy and Campbell 1994; Schulze et al. 2001; Burtis et al. 2016). Lone star ticks are remarkably mobile and will move toward host cues up to 10 m per day and 30 m over 2 days (Marshall et al. 2025). We observed substantially greater travel distances in the laboratory when tick movement was not obstructed by vegetation or ground litter structure. This capacity for movement is likely to influence the probability of tick–host contact, and it directly relates to tick detection when using traps baited with host cues (CO_2_) (Marshall et al. 2025). Although abundance and behavior have unique contributions to the likelihood of tick–host contact, they are typically conflated in field surveillance of ticks because the true abundance of ticks is unknown, and only active ticks are detected. Mark–recapture studies suggest that only a fraction of the tick population is active at any given time and tick activity is dynamic over time (Koch and McNew 1982; Kensinger and Allan 2011; Bugmyrin and Gorbach 2022; Marshall et al. 2025).

To examine how carryover effects on abundance and activity could jointly influence tick detection—a proxy for likelihood of tick–host contact—we simulated tick trapping events using abundance and activity measures from our laboratory experiments. We observed that carryover effects of temperature changed which population appeared to be more abundant over time. Early in the simulation (Days 1–5), ticks with a warm temperature history were detected at higher numbers compared to ticks with a cool temperature history, even though the relative abundances were similar for the two populations. This reflected increased movement of ticks with carryover effects of a warm environment (Figures 1 and 2) and highlights the importance of tick movement to detection and host contact. Later in the simulation (Days 10–20), ticks with a cool temperature history were detected at higher numbers. This reflected a combination of carryover effects on both mortality and behavior. Warm history ticks experienced higher mortality, which dropped their relative abundance below that of ticks with a cool history. In addition, warm history ticks moved less than cool history ticks in that later period, resulting in a lower probability of detection.

Our data suggest that near‐term carryover effects of temperature could affect how many ticks are detected in the environment as a function of mortality or behavior and illustrate that both tick movement and abundance are important for detection. However, the temperatures to which we exposed ticks were not fully realistic representations of conditions in nature. Although we used temperatures observed under field conditions (Cobos et al. 2024), we did not allow temperature or relative humidity to fluctuate as they naturally do over a 24‐h period. Under naturally fluctuating temperatures, it is possible that the carryover effects we observed would be dampened as the ticks experienced the temperature extremes transiently and could potentially recover from temperature effects on water loss (Bertrand and Wilson 1996; Crooks and Randolph 2006). Our experimental treatments were also limited to a relatively short period of time (4 weeks). Given the duration of tick activity in Oklahoma lasts up to 9 months, it is possible that thermal carryover effects could be magnified over longer periods of exposure, or effects may manifest over longer periods of time (i.e., months to years). These temporal aspects of carryover effects on tick ecology will require future research.

Another important avenue for future research is to determine the interactions between carryover effects of past temperature conditions and effects of concurrent temperature conditions (Vail and Smith 1998; Schulz et al. 2014; Portugal et al. 2020; Thomas et al. 2020). In their study of *I. ricinus* host‐seeking behavior across latitudinal clines, Gilbert et al. (2014) demonstrated that ticks are adapted to local thermal conditions and initiate questing at lower concurrent temperatures when the ticks have a cool thermal history relative to those from warmer climates. How carryover effects will manifest under a changing climate is unclear. Most climate change work focuses on changes in tick distribution (Raghavan et al. 2019; Gilbert 2021) and phenology (Levi et al. 2015; McClung et al. 2023) with the prediction that tick‐borne disease will increase due to shortened winters (Bouchard et al. 2019). Our work supports the possibility of a reduction in tick numbers in warming areas, potentially resulting in lower incidence of tick‐borne disease (Nuttall 2022). Improving our understanding of these phenomena will strengthen our ability to forecast tick‐borne disease (Ogden et al. 2008; Brugger et al. 2017; Cat et al. 2017). It is also possible that the temperature conditions could generate carryover effects on tick‐borne disease dynamics by altering pathogen prevalence (Saboret and Ingram 2019), vector density (Norris and Marra 2007), and vector competency (Barreaux et al. 2016). Further work is needed to describe these potential effects and their role in disease ecology.

## 5 Conclusion

Ambient temperature has carryover effects on the subsequent mortality rate and behavior of adult *A. americanum* . These effects alter tick abundance and the distances that ticks move, which are key parameters affecting tick detection using trapping as a surveillance tool. Our data suggest that temperature in the prior month may influence how many ticks are detected in the environment, which is important to estimating hazard for tick contact and tick‐borne pathogen transmission. Consideration of carryover effects may improve the accuracy of hazard forecasts and provide new insights into the ecology of tick‐borne disease.

## Author Contributions

**Daniel S. Marshall:** conceptualization (equal), data curation (lead), formal analysis (equal), funding acquisition (equal), investigation (lead), writing – original draft (lead), writing – review and editing (equal). **Karen C. Poh:** investigation (equal), software (equal), writing – review and editing (equal). **Jeb P. Owen:** conceptualization (equal), formal analysis (equal), supervision (lead), writing – review and editing (equal).

## Disclosure

*Statement on inclusion*: Our study includes authors from the country where the study was carried out. Authors are from disparate localities within the country, and all authors were engaged early with the research to ensure consideration of diverse perspectives in the study design. The lead author is a local researcher at the study location and led data collection at that site.

## Conflicts of Interest

The authors declare no conflicts of interest.

## Supporting information


**Data S1:** ece372252‐sup‐0001‐DataS1.zip.

## Data Availability

All data used to reproduce analyses can be found on Mendeley Data at https://data.mendeley.com/datasets/94bgxn33jt/1.

## References

[ece372252-bib-0001] Barnard, D. R. 1985. “Injury Thresholds and Production Loss Functions for the Lone Star Tick, *Amblyomma americanum* (Acari: Ixodidae), on Pastured, Preweaner Beef Cattle, *Bos taurus* .” Journal of Economic Entomology 78, no. 4: 852–855. 10.1093/jee/78.4.852. 4056195

[ece372252-bib-0003] Barnard, D. R. 1991. “Mechanisms of Host‐Tick Contact With Special Reference to *Amblyomma americanum* (Acari: Ixodidae) in Beef Cattle Forage Areas.” Journal of Medical Entomology 28, no. 5: 557–564. 10.1093/jmedent/28.5.557. 1941920

[ece372252-bib-0005] Barreaux, A. M. G. , P. Barreaux , K. Thievent , et al. 2016. “Larval Environment Influences Vector Competence of the Malaria Mosquito *Anopheles gambiae* .” MalariaWorld Journal 7, no. 8: 1–8. 10.5281/zenodo.10798340. 38601358 PMC11003208

[ece372252-bib-0006] Bates, D. , M. Mächler , B. Bolker , and S. Walker . 2015. “Fitting Linear Mixed‐Effects Models Using lme4.” Journal of Statistical Software 67, no. 1: 1–48. 10.18637/jss.v067.i01.

[ece372252-bib-0007] Benoit, J. B. , K. Oyen , G. Finch , et al. 2021. “Cold Hardening Improves Larval Tick Questing Under Low Temperatures at the Expense of Longevity.” Comparative Biochemistry and Physiology Part A: Molecular & Integrative Physiology 257: 110966. 10.1016/j.cbpa.2021.110966. PMC9936387 33895321

[ece372252-bib-0008] Bertrand, M. R. , and M. L. Wilson . 1996. “Microclimate‐Dependent Survival of Unfed Adult *Ixodes scapularis* (Acari: Ixodidae) in Nature: Life Cycle and Study Design Implications.” Journal of Medical Entomology 33, no. 4: 619–627. 10.1093/jmedent/33.4.619. 8699457

[ece372252-bib-0009] Bouchard, C. , A. Dibernardo , J. Koffi , et al. 2019. “N Increased Risk of Tick‐Borne Disease With Climate and Environmental Changes.” Canada Communicable Disease Report 45, no. 4: 83–89. 31285697 10.14745/ccdr.v45i04a02 PMC6587693

[ece372252-bib-0010] Brugger, K. , M. Walter , L. Chitimia‐Dobler , G. Dobler , and F. Rubel . 2017. “Seasonal Cycles of the TBE and Lyme Borreliosis Vector *Ixodes ricinus* Modelled With Time‐Lagged and Interval‐Averaged Predictors.” Experimental & Applied Acarology 73, no. 3–4: 439–450. 10.1007/s10493-017-0197-8. 29181672 PMC5727152

[ece372252-bib-0011] Bugmyrin, S. V. , and V. V. Gorbach . 2022. “Mark‐Release‐Recapture of Ticks: A Case Study of Estimating the Abundance of *Ixodes persulcatus* (Acari, Ixodidae).” Medical and Veterinary Entomology 36, no. 2: 185–193. 10.1111/mve.12565. 35122695

[ece372252-bib-0012] Burtis, J. C. , P. Sullivan , T. Levi , K. Oggenfuss , T. J. Fahey , and R. S. Ostfeld . 2016. “The Impact of Temperature and Precipitation on Blacklegged Tick Activity and Lyme Disease Incidence in Endemic and Emerging Regions.” Parasites & Vectors 9, no. 1: 606. 10.1186/s13071-016-1894-6. 27887625 PMC5124264

[ece372252-bib-0013] Burton, E. S. , R. S. Ostfeld , and J. L. Brunner . 2024. “Responses of Juvenile Blacklegged Ticks (Acari: Ixodidae) to Hosts of Varying Quality.” Journal of Medical Entomology 62, no. 1: 164–173. 10.1093/jme/tjae103. 39194343

[ece372252-bib-0014] Cat, J. , F. Beugnet , T. Hoch , F. Jongejan , A. Prangé , and K. Chalvet‐Monfray . 2017. “Influence of the Spatial Heterogeneity in Tick Abundance in the Modeling of the Seasonal Activity of *Ixodes ricinus* Nymphs in Western Europe.” Experimental and Applied Acarology 71, no. 2: 115–130. 10.1007/s10493-016-0099-1. 28127642

[ece372252-bib-0015] Cobos, M. E. , T. Winters , I. Martinez , et al. 2024. “Modeling Spatiotemporal Dynamics of *Amblyomma americanum* Questing Activity in the Central Great Plains.” PLoS One 19, no. 10: e0304427. 10.1371/journal.pone.0304427. 39466807 PMC11515986

[ece372252-bib-0016] Crooks, E. , and S. E. Randolph . 2006. “Walking by *Ixodes ricinus* Ticks: Intrinsic and Extrinsic Factors Determine the Attraction of Moisture or Host Odour.” Journal of Experimental Biology 209, no. 11: 2138–2142. 10.1242/jeb.02238. 16709915

[ece372252-bib-0017] Dobson, A. D. M. 2014. “History and Complexity in Tick‐Host Dynamics: Discrepancies Between ‘Real’ and ‘Visible’ Tick Populations.” Parasites & Vectors 7, no. 1: 231. 10.1186/1756-3305-7-231. 24885852 PMC4038084

[ece372252-bib-0018] Duffy, D. C. , and S. R. Campbell . 1994. “Ambient Air Temperature as a Predictor of Activity of Adult *Ixodes scapularis* (Acari: Ixodidae).” Journal of Medical Entomology 31, no. 1: 178–180. 10.1093/jmedent/31.1.178. 8158624

[ece372252-bib-0020] Eisen, R. J. , and C. D. Paddock . 2021. “Tick and Tickborne Pathogen Surveillance as a Public Health Tool in the United States.” Journal of Medical Entomology 58, no. 4: 1490–1502. 10.1093/jme/tjaa087. 32440679 PMC8905548

[ece372252-bib-0021] Falco, R. C. , and D. Fish . 1991. “Horizontal Movement of Adult *Ixodes dammini* (Acari: Ixodidae) Attracted to CO_2_‐Baited Traps.” Journal of Medical Entomology 28, no. 5: 726–729. 10.1093/jmedent/28.5.726. 1941943

[ece372252-bib-0022] Falco, R. C. , D. F. McKenna , T. J. Daniels , et al. 1999. “Temporal Relation Between *Ixodes scapularis* Abundance and Risk for Lyme Disease Associated With Erythema Migrans.” American Journal of Epidemiology 149, no. 8: 771–776. 10.1093/oxfordjournals.aje.a009886. 10206627

[ece372252-bib-0023] Festa‐Bianchet, M. 1998. “Condition‐Dependent Reproductive Success in Bighorn Ewes.” Ecology Letters 1, no. 2: 91–94. 10.1046/j.1461-0248.1998.00023.x.

[ece372252-bib-0024] Fieler, A. M. , A. J. Rosendale , D. W. Farrow , et al. 2021. “Larval Thermal Characteristics of Multiple Ixodid Ticks.” Comparative Biochemistry and Physiology. Part A, Molecular & Integrative Physiology 257: 110939. 10.1016/j.cbpa.2021.110939. PMC8500258 33794367

[ece372252-bib-0025] Gilbert, L. 2021. “The Impacts of Climate Change on Ticks and Tick‐Borne Disease Risk.” Annual Review of Entomology 66: 373–388. 10.1146/annurev-ento-052720-094533. 33417823

[ece372252-bib-0026] Gilbert, L. , J. Aungier , and J. L. Tomkins . 2014. “Climate of Origin Affects Tick ( *Ixodes ricinus* ) Host‐Seeking Behavior in Response to Temperature: Implications for Resilience to Climate Change?” Ecology and Evolution 4, no. 7: 1186–1198. 10.1002/ece3.1014. 24772293 PMC3997332

[ece372252-bib-0027] Gratton, C. , and R. F. Denno . 2003. “Inter‐Year Carryover Effects of a Nutrient Pulse on *Spartina* Plants, Herbivores, and Natural Enemies.” Ecology 84, no. 10: 2692–2707. 10.1890/02-0666.

[ece372252-bib-0028] Gunnarsson, T. G. , J. A. Gill , J. Newton , P. M. Potts , and W. J. Sutherland . 2005. “Seasonal Matching of Habitat Quality and Fitness in a Migratory Bird.” Proceedings of the Royal Society B: Biological Sciences 272, no. 1578: 2319–2323. 10.1098/rspb.2005.3214. PMC1560186 16191646

[ece372252-bib-0029] Halsey, L. G. , P. G. D. Matthews , E. L. Rezende , L. Chauvaud , and A. A. Robson . 2015. “The Interactions Between Temperature and Activity Levels in Driving Metabolic Rate: Theory, With Empirical Validation From Contrasting Ecotherms.” Oecologia 177, no. 4: 1117–1129. 25575673 10.1007/s00442-014-3190-5

[ece372252-bib-0030] Harrison, X. A. , J. D. Blount , R. Inger , D. R. Norris , and S. Bearhop . 2011. “Carry‐Over Effects as Drivers of Fitness Differences in Animals.” Journal of Animal Ecology 80, no. 1: 4–18. 10.1111/j.1365-2656.2010.01740.x. 20726924

[ece372252-bib-0031] Hopla, C. E. 1960. “The Transmission of Tularemia Organisms by Ticks in the Southern States.” Southern Medical Journal 53, no. 1: 92–97. 14403044 10.1097/00007611-196001000-00020

[ece372252-bib-0032] Jensen, P. M. 2000. “Host Seeking Activity of *Ixodes ricinus* Ticks Based on Daily Consecutive Flagging Samples.” Experimental & Applied Acarology 24, no. 9: 695–708. 10.1023/A:1010640219816. 11227827

[ece372252-bib-0033] Jongejan, F. , and G. Uilenberg . 2004. “The Global Importance of Ticks.” Parasitology 129, no. S1: S3–S14. 10.1017/s0031182004005967. 15938502

[ece372252-bib-0034] Kensinger, B. J. , and B. F. Allan . 2011. “Efficacy of Dry Ice‐Baited Traps for Sampling *Amblyomma americanum* (Acari: Ixodidae) Varies With Life Stage but Not Habitat.” Journal of Medical Entomology 48, no. 3: 708–711. 21661336 10.1603/me10275

[ece372252-bib-0035] Koch, H. G. 1984. “Survival of the Lone Star Tick, *Amblyomma americanum* (Acari: Ixodidae), in Contrasting Habitats and Different Years in Southeastern Oklahoma, USA.” Journal of Medical Entomology 21, no. 1: 69–79. 10.1093/jmedent/21.1.69.

[ece372252-bib-0036] Koch, H. G. , and R. W. McNew . 1982. “Sampling of Lone Star Ticks (Acari: Ixodidae): Dry Ice Quantity and Capture Success.” Annals of the Entomological Society of America 75, no. 5: 579–582. 10.1093/aesa/75.5.579.

[ece372252-bib-0037] Koch, H. G. , and M. D. Tuck . 1986. “Molting and Survival of the Brown Dog Tick (Acari: Ixodidae) Under Different Temperatures and Humidities.” Annals of the Entomological Society of America 79, no. 1: 11–14. 10.1093/aesa/79.1.11.

[ece372252-bib-0038] Lees, A. D. , and A. Milne . 1951. “The Seasonal and Diurnal Activities of Individual Sheep Ticks ( *Ixodes ricinus* L.).” Parasitology 41, no. 3–4: 189–208. 10.1017/S0031182000084031. 14911213

[ece372252-bib-0039] Levi, T. , F. Keesing , K. Oggenfuss , and R. S. Ostfeld . 2015. “Accelerated Phenology of Blacklegged Ticks Under Climate Warming.” Philosophical Transactions of the Royal Society, B: Biological Sciences 370, no. 1665: 20130556. 10.1098/rstb.2013.0556. PMC4342961 25688016

[ece372252-bib-0040] Levin, M. L. , G. E. Zemtsova , L. F. Killmaster , A. Snellgrove , and L. B. M. Schumacher . 2017. “Vector Competence of *Amblyomma americanum* (Acari: Ixodidae) for *Rickettsia rickettsii* .” Ticks and Tick‐Borne Diseases 8, no. 4: 615–622. 10.1016/j.ttbdis.2017.04.006. 28433728 PMC5657001

[ece372252-bib-0041] Marshall, D. S. , K. C. Poh , M. V. Reichard , L. A. Starkey , and J. P. Owen . 2025. “Spatial and Temporal Activity Patterns of *Amblyomma americanum* .” Parasites & Vectors 18: 12. 10.1186/s13071-025-06661-x. 39819362 PMC11740481

[ece372252-bib-0042] McClung, K. L. , K. D. Sundstrom , M. W. Lineberry , A. N. Grant , and S. E. Little . 2023. “Seasonality of *Amblyomma americanum* (Acari: Ixodidae) Activity and Prevalence of Infection With Tick‐Borne Disease Agents in North Central Oklahoma.” Vector‐Borne and Zoonotic Diseases 23, no. 11: 561–567. 10.1089/vbz.2023.0009. 37668606 PMC10654644

[ece372252-bib-0043] Mixson, T. R. , H. S. Ginsberg , S. R. Campbell , J. W. Sumner , and C. D. Paddock . 2004. “Detection of *Ehrlichia chaffeensis* in Adult and Nymphal *Amblyomma americanum* (Acari: Ixodidae) Ticks From Long Island, New York.” Journal of Medical Entomology 41, no. 6: 1104–1110. 10.1603/0022-2585-41.6.1104. 15605650

[ece372252-bib-0044] Nielebeck, C. , S. H. Kim , A. Pepe , et al. 2023. “Climatic Stress Decreases Tick Survival but Increases Rate of Host‐Seeking Behavior.” Ecosphere 14, no. 1: e4369. 10.1002/ecs2.4369.

[ece372252-bib-0045] Norris, D. R. 2005. “Carry‐Over Effects and Habitat Quality in Migratory Populations.” Oikos 109, no. 1: 178–186. 10.1111/j.0030-1299.2005.13671.x.

[ece372252-bib-0046] Norris, D. R. , and C. M. Taylor . 2006. “Predicting the Consequences of Carry‐Over Effects for Migratory Populations.” Biology Letters 2, no. 1: 148–151. 10.1098/rsbl.2005.0397. 17148350 PMC1617207

[ece372252-bib-0047] Norris, R. D. , and P. P. Marra . 2007. “Seasonal Interactions, Habitat Quality, and Population Dynamics in Migratory Birds.” Condor 109, no. 3: 535–547. 10.1093/condor/109.3.535.

[ece372252-bib-0048] Nuttall, P. A. 2022. “Climate Change Impacts on Ticks and Tick‐Borne Infections.” Biologia 77, no. 6: 1503–1512. 10.1007/s11756-021-00927-2.

[ece372252-bib-0049] Nuttall, P. A. , and M. Labuda . 1994. “Tick‐Borne Encephalitis Subgroup.” In Ecological Dynamics of Tick‐Borne Zoonoses, edited by D. E. Sonenshine and T. N. Mather . Oxford University Press. 10.1093/oso/9780195073133.003.0012.

[ece372252-bib-0050] O'Connor, C. M. , D. Norris , G. T. Crossin , D. R. Norris , and S. J. Cooke . 2014. “Biological Carryover Effects: Linking Common Concepts and Mechanisms in Ecology and Evolution.” Ecosphere 5, no. 3: 1–11. 10.1890/ES13-00388.1.

[ece372252-bib-0051] Ogden, N. H. , M. Bigras‐Poulin , K. Hanincová , A. Maarouf , C. J. O'Callaghan , and K. Kurtenbach . 2008. “Projected Effects of Climate Change on Tick Phenology and Fitness of Pathogens Transmitted by the North American Tick *Ixodes scapularis* .” Journal of Theoretical Biology 254, no. 3: 621–632. 10.1016/j.jtbi.2008.06.020. 18634803

[ece372252-bib-0052] Ogden, N. H. , L. R. Lindsay , G. Beauchamp , et al. 2004. “Investigation of Relationships Between Temperature and Developmental Rates of Tick *Ixodes scapularis* (Acari: Ixodidae) in the Laboratory and Field.” Journal of Medical Entomology 41, no. 4: 622–633. 10.1603/0022-2585-41.4.622. 15311453

[ece372252-bib-0053] Oliver, J. H. 1989. “Biology and Systematics of Ticks (Acari:Ixodida).” Annual Review of Ecology and Systematics 20, no. 1: 397–430. 10.1146/annurev.es.20.110189.002145.

[ece372252-bib-0054] Owen, J. P. , A. Vander Vliet , and G. A. Scoles . 2014. “Comparative Off‐Host Survival of Larval Rocky Mountain Wood Ticks ( *Dermacentor andersoni* ) Collected From Ecologically Distinct Field Populations: Survival of Larval Rocky Mountain Wood Ticks.” Medical and Veterinary Entomology 28, no. 3: 341–344. 10.1111/mve.12049. 24665893

[ece372252-bib-0055] Oyen, K. J. , L. Croucher , and J. B. Benoit . 2021. “Tonic Immobility Is Influenced by Starvation, Life Stage, and Body Mass in Ixodid Ticks.” Journal of Medical Entomology 58, no. 3: 1030–1040. 10.1093/jme/tjab003. 33590870 PMC8122239

[ece372252-bib-0056] Pepin, K. M. , R. J. Eisen , P. S. Mead , et al. 2012. “Geographic Variation in the Relationship Between Human Lyme Disease Incidence and Density of Infected Host‐Seeking *Ixodes scapularis* Nymphs in the Eastern United States.” American Journal of Tropical Medicine and Hygiene 86, no. 6: 1062–1071. 10.4269/ajtmh.2012.11-0630. 22665620 PMC3366524

[ece372252-bib-0057] Porter, C. H. , R. C. Collins , and A. D. Brandling‐Bennett . 1988. “Vector Density, Parasite Prevalence, and Transmission of *Onchocerca volvulus* in Guatemala.” American Journal of Tropical Medicine and Hygiene 39, no. 6: 567–574. 10.4269/ajtmh.1988.39.567. 3207177

[ece372252-bib-0058] Portugal, J. S. , R. Wills , and J. Goddard . 2020. “Laboratory Studies of Questing Behavior in Colonized Nymphal *Amblyomma maculatum* Ticks (Acari: Ixodidae).” Journal of Medical Entomology 57, no. 5: 1480–1487. 10.1093/jme/tjaa077. 32307540

[ece372252-bib-0059] R Core Team . 2023. “R: A Language and Environment for Statistical Computing.” https://www.r‐project.org/.

[ece372252-bib-0060] Raghavan, R. K. , A. T. Peterson , M. E. Cobos , R. Ganta , and D. Foley . 2019. “Current and Future Distribution of the Lone Star Tick, *Amblyomma americanum* (L.) (Acari: Ixodidae) in North America.” PLoS One 14, no. 1: e0209082. 10.1371/journal.pone.0209082. 30601855 PMC6314611

[ece372252-bib-0061] Randolph, S. E. , and K. Storey . 1999. “Impact of Microclimate on Immature Tick‐Rodent Host Interactions (Acari: Ixodidae): Implications for Parasite Transmission.” Journal of Medical Entomology 36, no. 6: 741–748. 10.1093/jmedent/36.6.741. 10593075

[ece372252-bib-0062] Rosendale, A. J. , M. E. Dunlevy , A. M. Fieler , D. W. Farrow , B. Davies , and J. B. Benoit . 2017. “Dehydration and Starvation Yield Energetic Consequences That Affect Survival of the American Dog Tick.” Journal of Insect Physiology 101: 39–46. 10.1016/j.jinsphys.2017.06.012. 28648807

[ece372252-bib-0063] Rosendale, A. J. , L. E. Romick‐Rosendale , M. Watanabe , M. E. Dunlevy , and J. B. Benoit . 2016. “Mechanistic Underpinnings of Dehydration Stress in the American Dog Tick Revealed Through RNA‐Seq and Metabolomics.” Journal of Experimental Biology 219, no. 12: 1808–1819. 10.1242/jeb.137315. 27307540

[ece372252-bib-0064] Rudolph, D. , and W. Knülle . 1974. “Site and Mechanism of Water Vapour Uptake From the Atmosphere in Ixodid Ticks.” Nature 249, no. 5452: 84–85. 10.1038/249084a0. 4833236

[ece372252-bib-0065] Saboret, G. , and T. Ingram . 2019. “Carryover Effects of Larval Environment on Individual Variation in a Facultatively Diadromous Fish.” Ecology and Evolution 9, no. 18: 10630–10643. 10.1002/ece3.5582. 31624571 PMC6787821

[ece372252-bib-0066] Savage, H. M. , K. L. Burkhalter , M. S. Godsey , et al. 2017. “Bourbon Virus in Field‐Collected Ticks, Missouri, USA.” Emerging Infectious Diseases 23, no. 12: 2017–2022. 10.3201/eid2312.170532. 29148395 PMC5708220

[ece372252-bib-0067] Savage, H. M. , M. S. Godsey , A. Lambert , et al. 2013. “First Detection of Heartland Virus (Bunyaviridae: *Phlebovirus*) From Field Collected Arthropods.” American Journal of Tropical Medicine and Hygiene 89, no. 3: 445–452. 10.4269/ajtmh.13-0209. 23878186 PMC3771279

[ece372252-bib-0068] Schulz, M. , M. Mahling , and K. Pfister . 2014. “Abundance and Seasonal Activity of Questing *Ixodes Ricinus* Ticks in Their Natural Habitats in Southern Germany in 2011.” Journal of Vector Ecology 39, no. 1: 56–65. 10.1111/j.1948-7134.2014.12070.x. 24820556

[ece372252-bib-0069] Schulze, T. L. , R. A. Jordan , and R. W. Hung . 1997. “Biases Associated With Several Sampling Methods Used to Estimate Abundance of *Ixodes scapularis* and *Amblyomma americanum* (Acari: Ixodidae).” Journal of Medical Entomology 34, no. 6: 615–623. 10.1093/jmedent/34.6.615. 9439115

[ece372252-bib-0070] Schulze, T. L. , R. A. Jordan , and R. W. Hung . 2001. “Potential Effects of Animal Activity on the Spatial Distribution of *Ixodes scapularis* and *Amblyomma americanum* (Acari: Ixodidae).” Environmental Entomology 30, no. 3: 568–577. 10.1603/0046-225X-30.3.568.

[ece372252-bib-0071] Semtner, P. J. , and J. A. Hair . 1973. “The Ecology and Behavior of the Lone Star Tick (Acarina: Ixodidae) V. Abundance and Seasonal Distribution in Different Habitat Types.” Journal of Medical Entomology 10, no. 6: 618–628. 10.1093/jmedent/10.6.618. 4779928

[ece372252-bib-0072] Sirén, A. P. K. , J. Berube , L. A. Clarfeld , C. F. Sullivan , B. Simpson , and T. L. Wilson . 2024. “Accounting for Missing Ticks: Use (Or Lack Thereof) of Hierarchical Models in Tick Ecology Studies.” Ticks and Tick‐Borne Diseases 15, no. 4: 102342. 10.1016/j.ttbdis.2024.102342. 38613901

[ece372252-bib-0073] Springer, Y. P. , L. Eisen , L. Beati , A. M. James , and R. J. Eisen . 2014. “Spatial Distribution of Counties in the Continental United States With Records of Occurrence of *Amblyomma americanum* (Ixodida: Ixodidae).” Journal of Medical Entomology 51, no. 2: 342–351. 10.1603/me13115. 24724282 PMC4623429

[ece372252-bib-0074] Stafford, K. C., III , G. Molaei , E. A. H. Little , C. D. Paddock , S. E. Karpathy , and A. M. Labonte . 2018. “Distribution and Establishment of the Lone Star Tick in Connecticut and Implications for Range Expansion and Public Health.” Journal of Medical Entomology 55, no. 6: 1561–1568. 10.1093/jme/tjy115. 30053108

[ece372252-bib-0075] Sutherst, R. W. , and A. S. Bourne . 2006. “The Effect of Desiccation and Low Temperature on the Viability of Eggs and Emerging Larvae of the Tick, *Rhipicephalus (Boophilus) Microplus* (Canestrini) (Ixodidae).” International Journal for Parasitology 36, no. 2: 193–200. 10.1016/j.ijpara.2005.09.007. 16300766

[ece372252-bib-0077] Therneau, T. M. , T. Lumley , and A. Elizabeth . 2024. “Survival: Survival Analysis.” https://cran.r‐project.org/web/packages/survival/index.html.

[ece372252-bib-0078] Thomas, C. E. , E. S. Burton , and J. L. Brunner . 2020. “Environmental Drivers of Questing Activity of Juvenile Black‐Legged Ticks (Acari: Ixodidae): Temperature, Desiccation Risk, and Diel Cycles.” Journal of Medical Entomology 57, no. 1: 8–16. 10.1093/jme/tjz126. 31370063

[ece372252-bib-0079] Troughton, D. R. , and M. L. Levin . 2007. “Life Cycles of Seven Ixodid Tick Species (Acari: Ixodidae) Under Standardized Laboratory Conditions.” Journal of Medical Entomology 44, no. 5: 732–740. 10.1093/jmedent/44.5.732. 17915502

[ece372252-bib-0080] Vail, S. G. , and G. Smith . 1998. “Air Temperature and Relative Humidity Effects on Behavioral Activity of Blacklegged Tick (Acari: Ixodidae) Nymphs in New Jersey.” Journal of Medical Entomology 35, no. 6: 1025–1028. 10.1093/jmedent/35.6.1025. 9835697

[ece372252-bib-0081] Vail, S. G. , and G. Smith . 2002. “Vertical Movement and Posture of Blacklegged Tick (Acari: Ixodidae) Nymphs as a Function of Temperature and Relative Humidity in Laboratory Experiments.” Journal of Medical Entomology 39, no. 6: 842–846. 10.1603/0022-2585-39.6.842. 12495181

[ece372252-bib-0082] Van Allen, B. G. , and V. H. W. Rudolf . 2016. “Carryover Effects Drive Competitive Dominance in Spatially Structured Environments.” Proceedings of the National Academy of Sciences 113, no. 25: 6939–6944. 10.1073/pnas.1520536113. PMC4922188 27298356

[ece372252-bib-0083] van Es, R. P. , J. E. Hillerton , and G. Gettinby . 1998. “Lipid Consumption in *Ixodes ricinus* (Acari: Ixodidae): Temperature and Potential Longevity.” Bulletin of Entomological Research 88, no. 5: 567–573. 10.1017/S0007485300026092.

[ece372252-bib-0084] Van Gestel, M. , D. Heylen , K. Verheyen , et al. 2024. “Recreational Hazard: Vegetation and Host Habitat Use Correlate With Changes in Tick‐Borne Disease Hazard at Infrastructure Within Forest Stands.” Science of the Total Environment 919: 170749. 10.1016/j.scitotenv.2024.170749. 38340833

[ece372252-bib-0085] Walk, S. T. , G. Xu , J. W. Stull , and S. M. Rich . 2009. “Correlation Between Tick Density and Pathogen Endemicity, New Hampshire.” Emerging Infectious Diseases 15, no. 4: 585–587. 10.3201/eid1504.080940. 19331738 PMC2671416

[ece372252-bib-0086] Walter, K. S. , K. M. Pepin , C. T. Webb , et al. 2016. “Invasion of Two Tick‐Borne Diseases Across New England: Harnessing Human Surveillance Data to Capture Underlying Ecological Invasion Processes.” Proceedings of the Royal Society B: Biological Sciences 283, no. 1832: 20160834. 10.1098/rspb.2016.0834. PMC4920326 27252022

[ece372252-bib-0087] Wilson, M. L. , E. A. Dykstra , and B. A. Schmidt . 1993. “Temperature‐ and Humidity‐Dependent Longevity of Unfed Adult *Hyalomma truncatum* (Acari: Ixodidae).” Journal of Medical Entomology 30, no. 2: 467–471. 10.1093/jmedent/30.2.467. 8459425

[ece372252-bib-0088] Zahler, M. , and R. Gothe . 1995. “Effect of Temperature and Humidity on Longevity of Unfed Adults and on Oviposition of Engorged Females of *Dermacentor reticulatus* (Ixodidae).” Applied Parasitology 36, no. 3: 200–211. 8541893

[ece372252-bib-0089] Zając, Z. , J. Kulisz , A. Woźniak , et al. 2023. “Tick Activity, Host Range, and Tick‐Borne Pathogen Prevalence in Mountain Habitats of the Western Carpathians, Poland.” Pathogens 12, no. 9: 1186. 10.3390/pathogens12091186. 37764994 PMC10534405

