# Supplementary material for: Carryover Effects of Thermal Conditions on Tick Survival, Behavior, and Simulated Detectability
Source: Ecol Evol. 2025 Oct 2;15(10):e72252. doi: 10.1002/ece3.72252 (PMC12490961; doi:10.1002/ece3.72252)

**Supplementary figures.**

Supplemental figure 1. Image of the *Amblyomma americanum* behavioural bioassay set-up. Adult ticks were contained in petri dishes resting on an overturned aquarium. Inside the aquarium was a ring light with sheets of mylar to diffuse light. The camera was mounted above the petri dishes so that ticks were lit from below to prevent glare. Photo credit: D.S. Marshall.


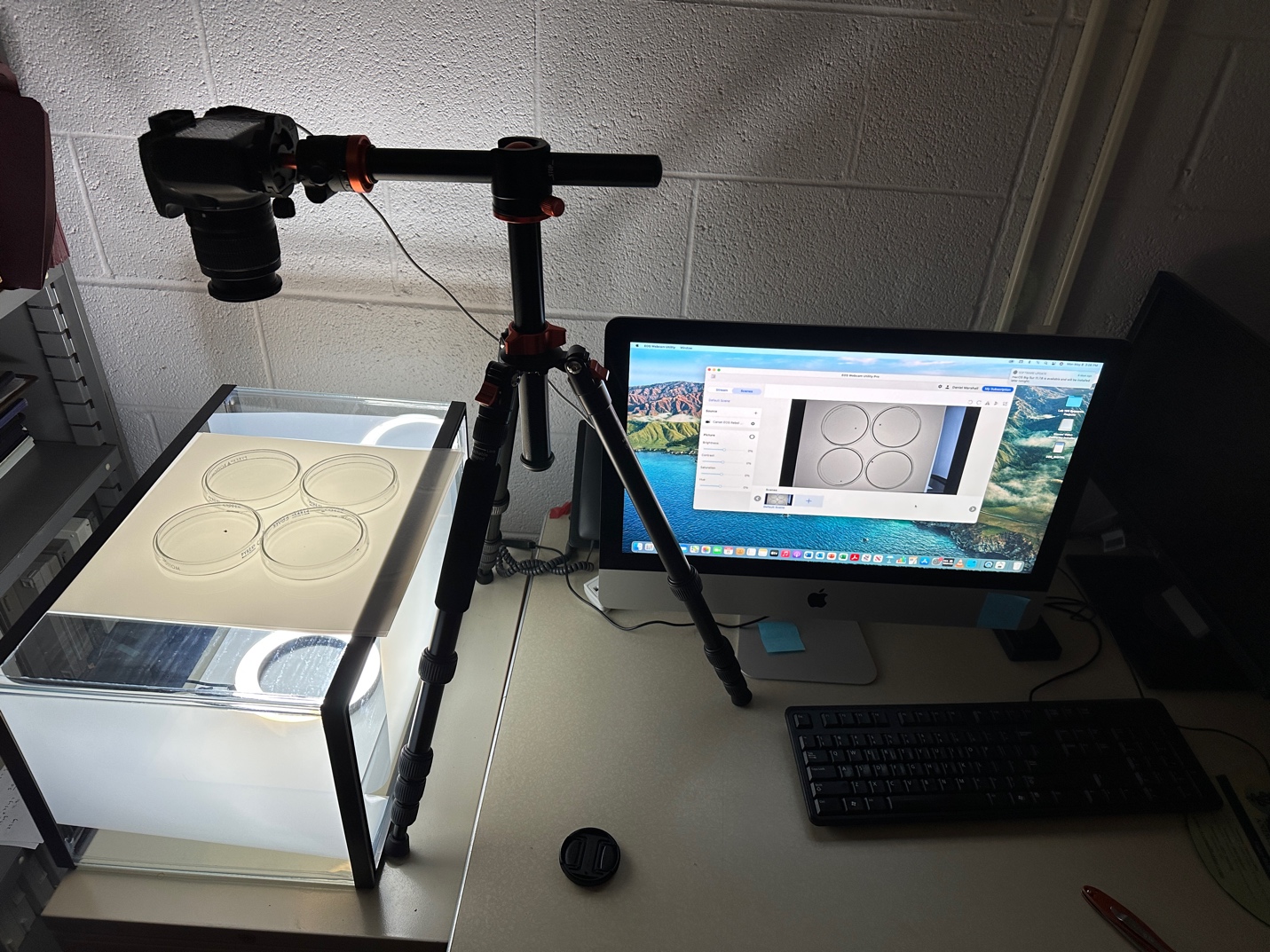


Supplemental figure 2. Proportion of adult *Amblyomma americanum* alive over 35 days after 4-week exposure to warm conditions. Points represent simulated values and the shaded region represents the 99% confidence interval for survival probability observed with experimental ticks. The shaded region only extends over the period in which survival varied for experimental ticks.


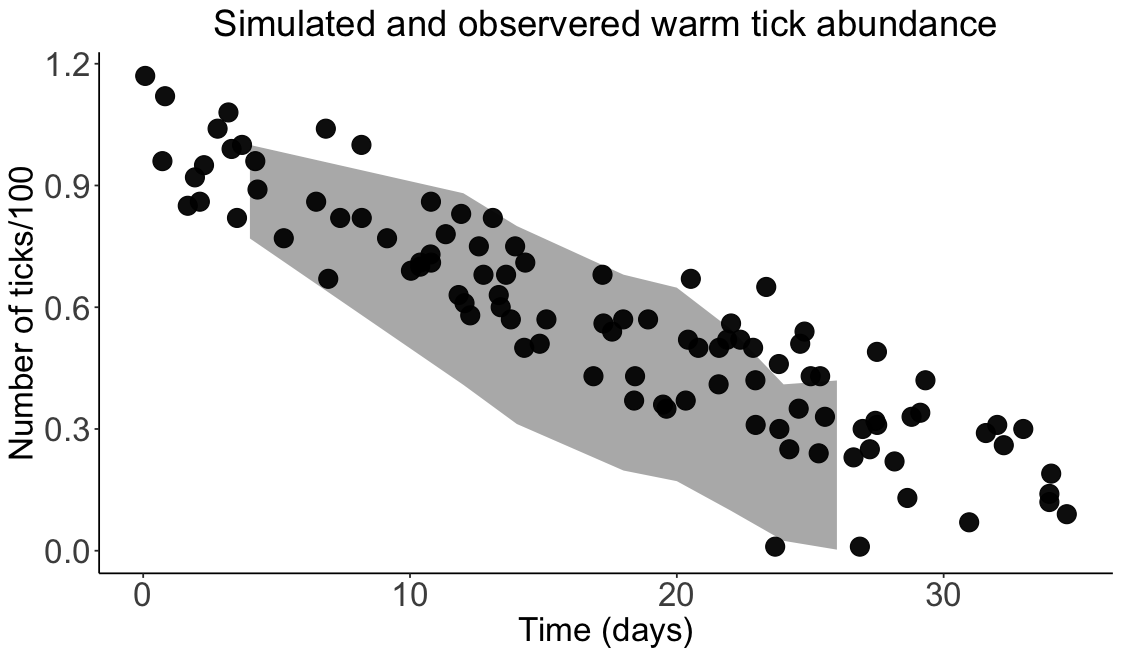


Supplemental figure 3. Supplemental figure 2. Proportion of adult *Amblyomma americanum* alive over 35 days after 4-week exposure to cool conditions. Points represent simulated values and the shaded region represents the 99% confidence interval for survival probability observed with experimental ticks. The shaded region only extends over the period in which survival varied for experimental ticks.


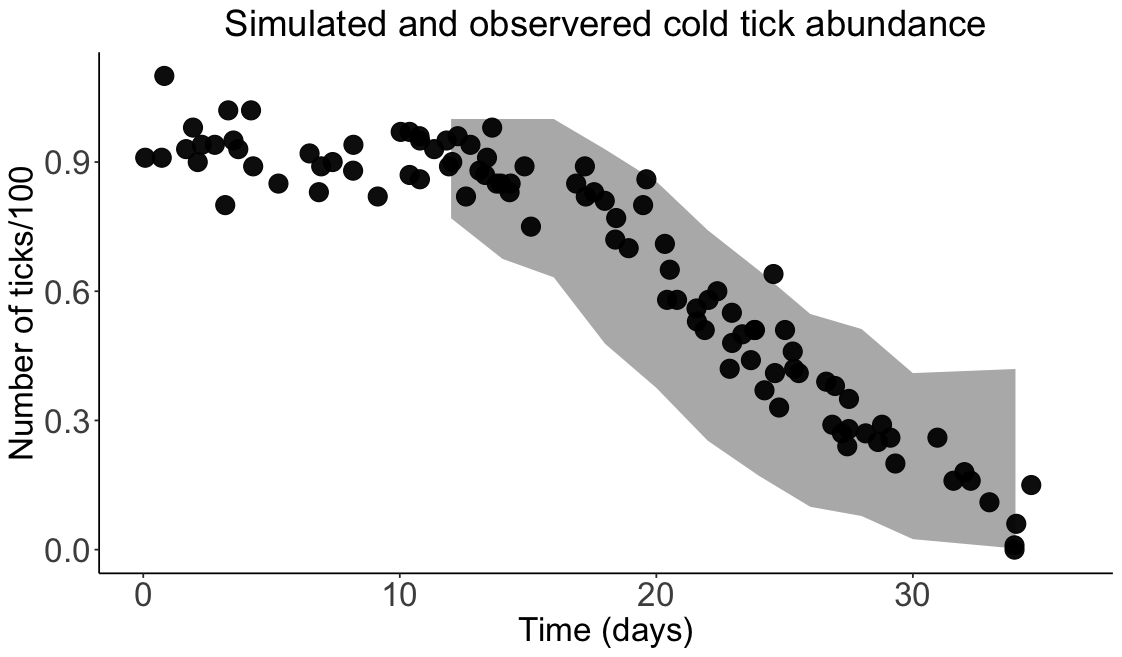


Supplemental figure 4. The simulated abundances of adult *Amblyomma americanum* over 35 days after 4-week exposure to warm (dark grey) and cool (light grey) temperatures.


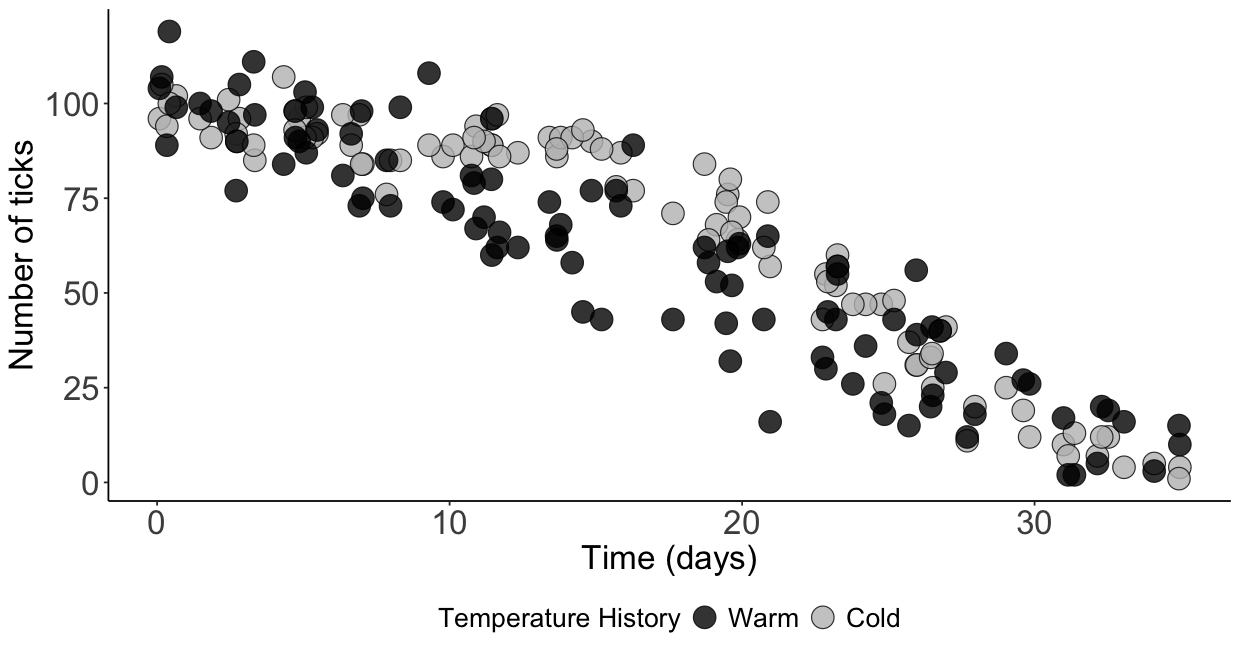


Supplementary figure 5. Observed (black) and simulated (grey) distances moved by adult *Amblyomma americanum* over 24-hour periods within 35 days following exposure to warm conditions (35 °C, 90% relative humidity, 14:10 light:dark cycle).


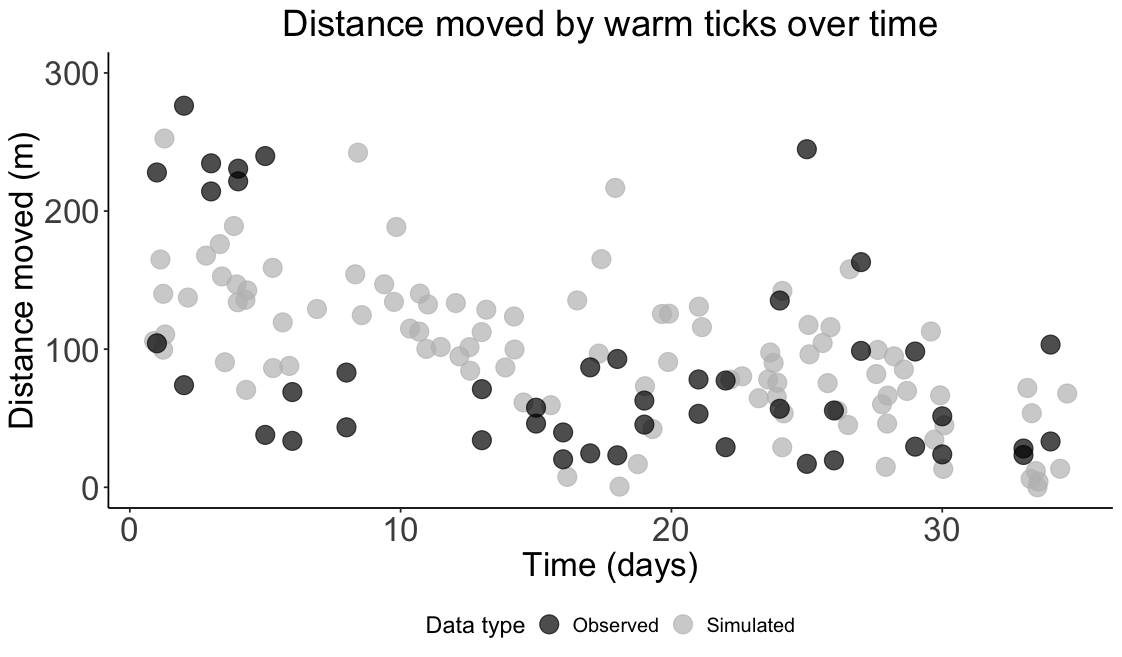


Supplementary figure 6. Observed (black) and simulated (grey) distances moved by adult *Amblyomma americanum* over 24-hour periods within 35 days following exposure to cool conditions (35 °C, 90% relative humidity, 14:10 light:dark cycle).


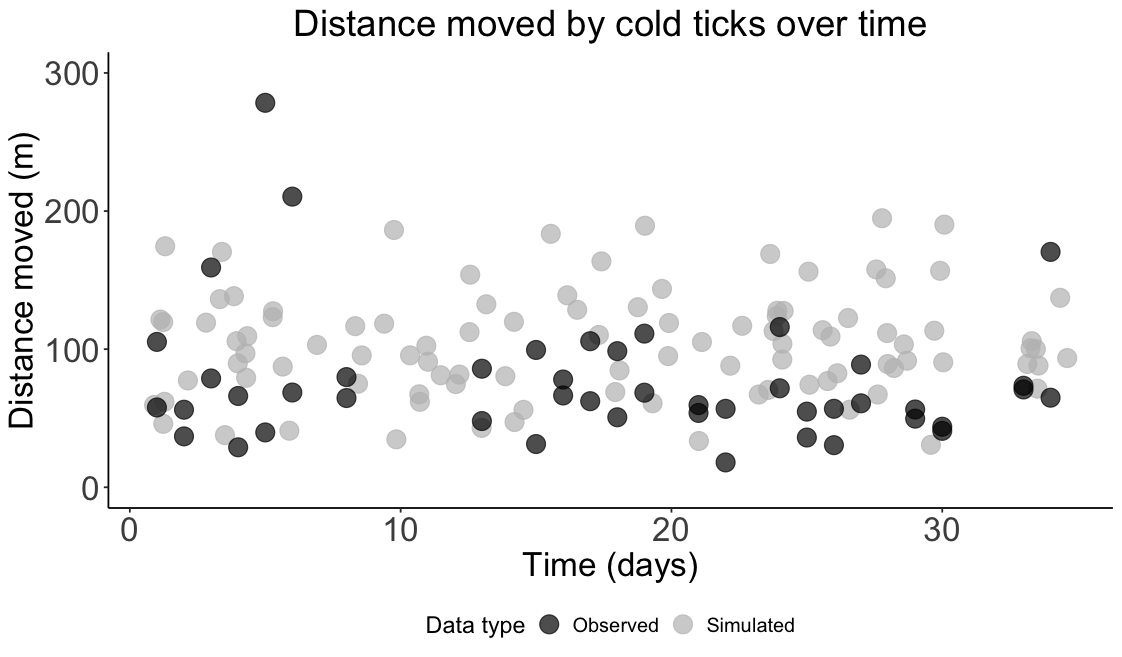


Supplementary figure 7. The detection probability relative to simulated distances travelled by adult *Amblyomma americanum* with a warm temperature history.


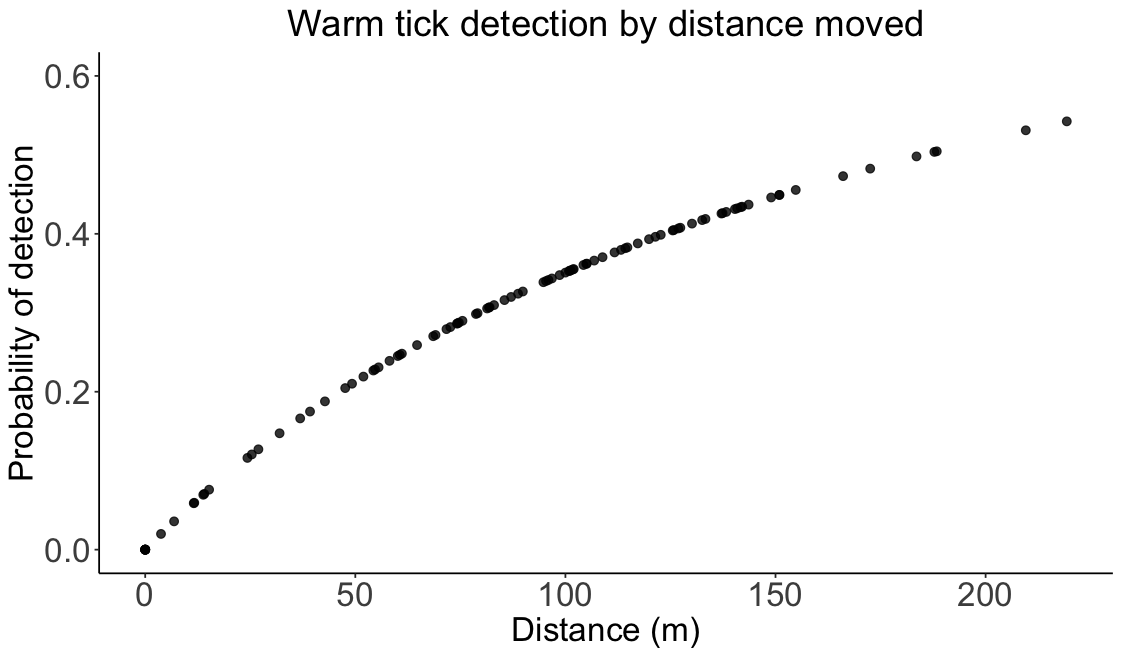


Supplementary figure 8. The detection probability relative to simulated distances travelled by adult *Amblyomma americanum* with a cool temperature history.


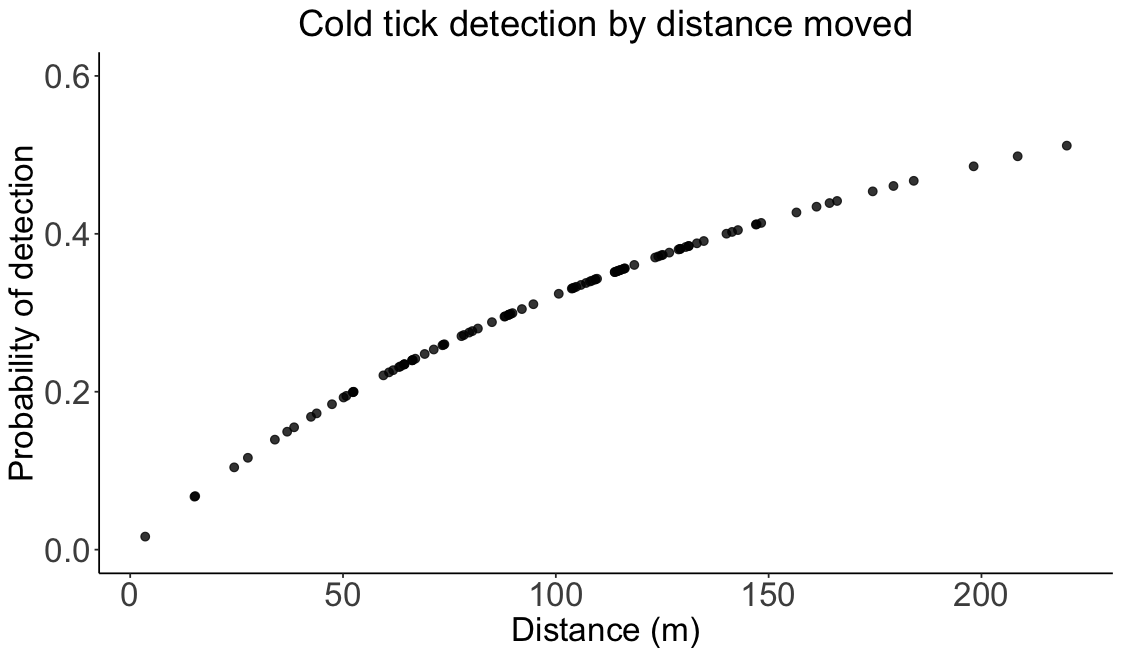

Supplement: Supplementary file 1 — Data S1: ece372252‐sup‐0001‐DataS1.zip. [file ECE3-15-e72252-s001.zip › ece372252-sup-0001-Figures.docx]
